# Age-Related Trends in Dual-Energy X-Ray Absorptiometry–Measured Adiposity and Their Clinical Relevance: A Multicenter Cross-Sectional Study of Korean Peri- and Postmenopausal Women

**DOI:** 10.3390/medicina61071301

**Published:** 2025-07-19

**Authors:** Jung Yoon Park, Hyoung Moo Park, Youn-Jee Chung, Mee-Ran Kim, Kyung Jin Hwang, Jae-Yen Song

**Affiliations:** 1 Department of Obstetrics and Gynecology, Seoul St. Mary’s Hospital, College of Medicine, The Catholic University of Korea, Seoul 06591, Republic of Korea; aurorix86@naver.com (J.Y.P.); porshe80@catholic.ac.kr (Y.-J.C.); drmrkim@gmail.com (M.-R.K.); 2 Department of Obstetrics and Gynecology, Menopause Clinic Grace Women’s Hospital, Goyang 10444, Republic of Korea; hmpark52@hanmail.net (H.M.P.); drhkj@naver.com (K.J.H.)

**Keywords:** body composition, adiposity, menopause, abdominal fat, absorptiometry

## Abstract

*Background and Objectives:* Body composition changes with aging and menopause, often leading to increased adiposity and a shift in fat distribution. While BMI is commonly used in clinical practice, it does not accurately reflect fat mass or distribution. This study aims to evaluate age-related changes in both total and regional adiposity using DXA-derived indices in Korean women aged ≥ 40 years and to assess the limitations of BMI-based obesity classification. *Materials and Methods:* This retrospective multicenter study analyzed the DXA scans and clinical records of 914 Korean women aged 40–80 years who attended menopause clinics across multiple institutions between 2018 and 2021. We analyzed five adiposity indices: body mass index (BMI), total body fat percentage (TB%F), fat mass index (FMI), visceral adipose tissue (VAT) area, and android-to-gynoid (A/G) fat ratio. Excess adiposity was defined as BMI ≥ 23 kg/m^2^, TB%F ≥ 40%, FMI ≥ 9 kg/m^2^, VAT > 100 cm^2^, or A/G ratio > 1.0. Age group comparisons were made using ANOVA, and misclassification was assessed by comparing BMI with other indices. *Results*: Mean BMI increased with age, peaking in the 60s before declining in the 70s. TB%F and FMI peaked in the 50s, while VAT and A/G ratio increased continuously with age. Excess adiposity was found in 41.9% of women by TB%F, 40.5% by FMI, and 59.4% by VAT in the 70s. Notably, 22% of women with normal BMI (<23 kg/m^2^) had VAT > 100 cm^2^, and 35.7% had A/G > 1.0, indicating central obesity. *Conclusions*: DXA-based indices provide a more accurate assessment of adiposity and associated cardiometabolic risks in aging women than BMI alone. Clinical screening strategies should consider incorporating regional fat distribution markers, particularly in midlife and postmenopausal populations, to better identify individuals at risk.

## 1. Introduction

Obesity is a multifactorial health issue with rising global prevalence, especially in aging populations [1,2]. In women, the menopausal transition accelerates changes in body composition, including an increase in fat mass and a redistribution toward central fat accumulation. These alterations are driven by hormonal shifts, reduced energy expenditure, and lifestyle factors [3,4].

### 1.1. Biological and Hormonal Mechanisms of Aging in Women

Midlife represents a critical period during which biological aging and hormonal changes converge to influence body composition and metabolic health. As part of the physiological aging process, adults typically experience a gradual decline in organ and tissue function, with estimates suggesting a reduction of up to 2% annually. This decline contributes to a progressive loss of skeletal muscle mass and a concomitant increase in adipose tissue, even in the absence of overt disease [5]. In young adults, skeletal muscle turnover—comprising both protein synthesis and degradation—is tightly regulated to preserve lean body mass [6,7]. However, aging disrupts this balance. Muscle mass, which accounts for approximately 30% of total body protein turnover in youth, may decline to below 20% in older adults. Beginning around the age of 50, skeletal muscle mass decreases by an estimated 1–2% per year, a trajectory that may accelerate with sedentary behavior or chronic disease [8,9].

Longitudinal analyses indicate that between ages 30 and 60, adults may gain roughly 0.45 kg of fat and lose 0.23 kg of lean mass per year. Over several decades, this pattern can culminate in a net increase of over 14 kg in fat and a loss exceeding 6 kg in muscle, contributing to a body composition profile increasingly characterized by sarcopenic adiposity [10]. In women, these age-related changes are compounded by the hormonal alterations of menopause. The sharp decline in estrogen during the menopausal transition facilitates a shift in fat storage patterns—from predominantly subcutaneous depots to increased visceral adipose tissue (VAT) accumulation [11]. This redistribution has important clinical implications, as the proportion of VAT typically rises from 5–8% of total fat mass in premenopausal women to 15–20% following menopause [12].

These physiological alterations have important clinical consequences, as women in midlife are particularly vulnerable to cardiometabolic complications associated with obesity. Epidemiological data suggest that the burden of obesity is disproportionately higher in women than in men; for example, findings from the National Health and Nutrition Examination Survey (NHANES) report a higher prevalence of obesity among women (40.4%) compared to men (35.0%) in the United States [13]. While midlife weight gain has traditionally been regarded as a natural consequence of chronological aging, evidence from the longitudinal Study of Women’s Health Across the Nation (SWAN), which followed more than 3300 women, indicates that hormonal shifts—particularly the decline in circulating estrogen—play a pivotal role in driving fat redistribution and adverse changes in body composition during the menopausal transition [14].

### 1.2. Limitations of BMI and the Clinical Value of DXA in Midlife Women

Menopause is now recognized as a pivotal period in women’s metabolic health, during which the decline in estrogen contributes to profound changes in adipose tissue biology [15]. Postmenopausal women experience a shift from subcutaneous to visceral fat deposition, along with adipocyte hypertrophy, chronic low-grade inflammation, and impaired insulin signaling [16,17]. This adipose tissue remodeling, characterized by increased macrophage infiltration and pro-inflammatory cytokine release, promotes systemic metabolic dysfunction [18]. These alterations not only increase total fat mass but also disproportionately affect the quality and distribution of fat, thereby increasing the risk of cardiometabolic diseases such as type 2 diabetes, hypertension, and atherosclerosis—even in women with normal BMI [19].

Central obesity, particularly the accumulation of visceral adipose tissue (VAT), is strongly associated with adverse cardiometabolic outcomes [20]. Traditionally, obesity has been assessed using body mass index (BMI), a simple and widely used metric derived from weight and height [21,22]. However, BMI does not differentiate between fat and lean mass, nor does it capture regional fat distribution—especially VAT—which has a closer relationship with metabolic risk [23].

Dual-energy X-ray absorptiometry (DXA) is a validated method for assessing body composition that offers accurate and reproducible measurements of total and regional adiposity. DXA-derived indices, including total body fat percentage (TB%F), fat mass index (FMI), VAT area, and android-to-gynoid (A/G) fat ratio, provide a more nuanced view of adiposity and its health implications [24,25].

Notably, there is a lack of population-based data examining how DXA-derived adiposity indices change with age in Asian women, particularly in the Korean population [26,27]. Understanding these age-related trends is critical for identifying women at elevated cardiometabolic risk who may be misclassified by BMI-based criteria alone [28].

Therefore, the primary aim of this study was to investigate age-related trends in body fat distribution using DXA-derived adiposity indices—including VAT, A/G ratio, TB%F, and FMI—in Korean women aged 40 years and older. The secondary aim was to examine the discordance between BMI-based obesity classification and DXA-derived adiposity patterns, with particular emphasis on identifying women with metabolically unfavorable fat distribution despite normal BMI. These objectives aim to inform more accurate risk stratification strategies and support the integration of advanced adiposity indices into routine clinical assessment for aging women.

## 2. Methods

This multicenter retrospective cross-sectional study analyzed 914 women aged 40–80 years who underwent DXA scans between June 2018 and June 2021 at a university hospital and a regional medical hospital in Korea. All women had visited the menopause clinic for routine evaluation or health screening. Exclusion criteria included a history of malignancy, autoimmune disease, or any chronic illness known to affect body composition.

This study adhered to the STROBE (Strengthening the Reporting of Observational Studies in Epidemiology) guidelines for cross-sectional studies. The STROBE checklist has been submitted as a supplementary file [29].

Height and weight were measured using standardized protocols, and BMI was calculated as weight (kg) divided by height (m^2^) [30]. Whole-body DXA scans were performed using a Hologic DXA system (Hologic Inc., Marlborough, MA, USA) with Horizon software for analysis. All participating institutions used the same model of DXA device and identical software versions to ensure methodological consistency. All DXA scans were conducted by trained radiologic technologists following a standardized protocol across both sites to ensure consistency. Centralized calibration and quality assurance procedures were performed regularly to minimize inter-institutional variability. The following indices were derived:

The adiposity indices assessed in this study included total body fat percentage (TB%F), fat mass index (FMI), visceral adipose tissue (VAT) area, and the android-to-gynoid (A/G) fat ratio. Total body fat percentage reflects the proportion of an individual’s weight composed of fat mass. The fat mass index (FMI) is calculated by dividing the total fat mass in kilograms by the square of the individual’s height in meters (kg/m^2^), offering a height-adjusted measure of adiposity [25]. The VAT area, measured in square centimeters, quantifies the amount of visceral fat—fat stored within the abdominal cavity, surrounding internal organs [24]. The A/G fat ratio represents the distribution of body fat between the android (abdominal) and gynoid (hip and thigh) regions, with higher values indicating a greater degree of central (abdominal) fat accumulation [31].

Excess adiposity was defined based on established clinical thresholds. Overweight and obesity were defined as a BMI of ≥23 kg/m^2^ and ≥25 kg/m^2^, respectively, according to the WHO Asia-Pacific and Korean Society for the Study of Obesity (KSSO) guidelines [32]. A TB%F of ≥40% was considered indicative of excess fat, as suggested by Dufour et al. [33,34,35]. An FMI of ≥9 kg/m^2^, as proposed by Kelly et al., was used to define elevated fat mass [25]. Central obesity was defined as a VAT area greater than 100 cm^2^ [24], and an A/G fat ratio exceeding 1.0 was considered indicative of an android or abdominal fat distribution pattern [36,37].

Statistical analysis was performed using SPSS version 23.0 (IBM, Armonk, NY, USA). Between-group comparisons used ANOVA with Bonferroni correction; chi-square tests compared prevalence across age groups. Statistical significance was defined as *p* < 0.05.

## 3. Results

A total of 917 Korean women aged 40 years and older were included in the analysis. Participants were categorized into four age groups: 40–49 years (*n* = 128), 50–59 years (*n* = 545), 60–69 years (*n* = 209), and ≥70 years (*n* = 35). The mean age was 56.2 years. The overall mean height was 157.7 cm, the mean body weight was 56.2 kg, and the mean body mass index (BMI) was 22.8 kg/m^2^ (Table 1).

DXA-derived adiposity indices showed distinct patterns according to age. Mean BMI increased with age and peaked in the 60s (23.6 kg/m^2^), followed by a slight decrease in the 70s (22.8 kg/m^2^). TB%F was highest in the 50s (39.0%), with a mild decline thereafter. Fat mass index (FMI) demonstrated a similar peak in the 50s (8.85 kg/m^2^). In contrast, visceral adipose tissue (VAT) and android-to-gynoid fat ratio (A/G ratio) increased consistently with age, peaking in the 70s at 107.6 cm^2^ and 1.06, respectively (Table 2).

Excess adiposity was prevalent across all age groups. The proportion of overweight and obese individuals (BMI ≥ 23 kg/m^2^) was 38.8%, with the highest prevalence in the 60–69 age group (44.5%). A TB%F ≥ 40% was observed in 41.9% of participants, most commonly in women in their 50s (44.6%). FMI ≥ 9 kg/m^2^ was found in 37.6% of the cohort, with the highest prevalence in the 60s (43.5%). The proportion of participants with VAT > 100 cm^2^ increased steadily with age, from 22.6% in the 40s to 48.5% in the 70s. An A/G ratio > 1.0, indicative of central adiposity, was observed in 49.2% of participants and showed a progressive increase across age groups, from 31.3% in the 40s to 60.0% in the 70s.

A more detailed breakdown of high-risk adiposity indices by age group is presented in Table 3. The highest proportion of women with TB%F ≥ 40% was observed in the 50s (44.6%), whereas FMI ≥ 9 kg/m^2^ peaked in the 60s (43.5%). VAT >100 cm^2^ increased consistently with age, suggesting age-related accumulation of visceral fat. The detailed adiposity outcomes for each individual by age in the total cohort are provided in Supplementary Data S1.

### 3.1. Adiposity Characteristics According to BMI Categories in Women over Age 40

In this study, we analyzed adiposity indices including total body fat percentage (TB%F), fat mass index (FMI), visceral adipose tissue (VAT), and android-to-gynoid (A/G) fat ratio according to BMI categories in Korean women aged 40 years and older (Table 4).

#### 3.1.1. General Trends in Body Fat Accumulation (TB%F, FMI)

Both TB%F and FMI demonstrated a progressive increase across BMI categories. Underweight women had a mean TB%F of 32.2% and an FMI of 5.6 kg/m^2^, while those with obesity (BMI ≥ 25 kg/m^2^) had markedly elevated values (TB%F 43.0%, FMI 11.3 kg/m^2^). The severely obese group (BMI ≥ 30 kg/m^2^) showed the highest TB%F (47.9%) and FMI (13.6 kg/m^2^), with standard deviations indicating greater variability within this group. These findings suggest that while total adiposity increases with BMI, the variance in fat composition becomes more prominent in the highest BMI category.

#### 3.1.2. Visceral Fat Accumulation (VAT Stratification)

VAT distribution varied significantly across BMI categories. Notably, 100% of underweight women and 78.0% of those with normal BMI had VAT < 100 cm^2^ (classified as “normal”). However, among overweight women, only 31.2% remained in this normal range, while 62.8% had VAT between 100 and 160 cm^2^ (borderline), and 6.0% exceeded 160 cm^2^ (high risk). In the obese group, the prevalence of VAT > 160 cm^2^ rose dramatically to 36.9%. These findings highlight that even within the overweight category, visceral adiposity becomes prominent and potentially harmful.

#### 3.1.3. Central Fat Distribution Pattern (A/G Ratio and Body Shape)

An age-related and BMI-dependent shift from gynoid (pear-shaped) to android (apple-shaped) fat distribution was clearly observed. Among women with normal BMI, 35.7% had an android body shape. This proportion increased to 67.3% in overweight women and further to 82.8% in those classified as obese. In contrast, pear-shaped distribution was dominant in the underweight (91.7%) and normal BMI groups (64.3%) but diminished with increasing BMI.

In summary, while BMI, TB%F, and FMI peaked during midlife and plateaued or declined thereafter, VAT and A/G ratio continued to increase into older age. These findings highlight the progressive nature of central fat accumulation with aging and the need for comprehensive adiposity assessment beyond BMI in postmenopausal women.

## 4. Discussion

This study investigated age-related changes in adiposity patterns among Korean women aged 40 years and older using DXA-derived body composition indices. The findings demonstrate that while general adiposity indices such as BMI, TB%F, and FMI tend to peak during midlife (50s–60s), regional adiposity markers, particularly VAT and A/G ratio, show a continuous upward trend into older age. This progressive increase in central adiposity with age has significant implications for cardiometabolic health in postmenopausal women [19].

The results are consistent with previous studies suggesting that the menopausal transition accelerates the redistribution of fat from peripheral to central compartments, driven by estrogen deficiency, changes in energy balance, and age-related metabolic decline [3,15]. Notably, our study revealed that nearly half of all participants had an A/G ratio > 1.0 and that VAT > 100 cm^2^ was observed in over 40% of the cohort. These proportions increased markedly with age, underscoring the need to evaluate regional fat accumulation as women age [24].

An important observation was the discrepancy between BMI and DXA-derived adiposity indices. A substantial proportion of women classified as having normal BMI (<23 kg/m^2^) demonstrated excess VAT and elevated A/G ratios, suggesting the presence of metabolically unfavorable fat distribution that may not be detected through BMI alone. These findings reinforce concerns that reliance on BMI may lead to the underestimation of central obesity and associated health risks, particularly in aging Asian women who may exhibit normal-weight obesity (NWO) phenotypes [19,24].

Furthermore, the strong correlation between general obesity (BMI ≥ 25 kg/m^2^) and A/G ratio > 1.0 in over 80% of participants in this category highlights that central adiposity commonly accompanies overt obesity. However, its presence in normal-weight individuals warrants targeted screening using DXA or other imaging modalities, especially for preventive cardiometabolic risk stratification [38].

The continuous rise in VAT and A/G ratio into the 70s contrasts with the plateau observed in BMI and TB%F, suggesting that age-related metabolic risk increases may be more closely associated with fat distribution than with absolute fat mass [39,40]. These findings support the incorporation of regional adiposity indices in routine assessment, particularly for older women who may be misclassified by conventional BMI-based criteria [41].

### 4.1. Clinical Implications and Integration with Updated Obesity Guidelines

The observed age-related increase in central adiposity—particularly in VAT and the android-to-gynoid (A/G) fat ratio—has critical implications for clinical screening and intervention strategies in midlife and older women. These regional fat indices revealed substantial proportions of women with metabolically adverse fat profiles despite normal BMI classifications. Notably, 22% of women with BMI < 23 kg/m^2^ had VAT > 100 cm^2^, and 35.7% had A/G ratio > 1.0, highlighting the presence of hidden central adiposity. These findings suggest that traditional reliance on BMI alone may overlook women at high cardiometabolic risk due to normal-weight obesity (NWO), especially prevalent in Asian populations [42,43].

To address this diagnostic gap, our study aligns with the 2025 definition of obesity recently proposed by The Lancet Diabetes & Endocrinology Commission, which introduces a paradigm shift from BMI-centric classification to a function- and organ-impairment-based approach [44]. This framework distinguishes between preclinical obesity—characterized by excess adiposity without overt metabolic dysfunction—and clinical obesity, where functional limitations or organ impairment are present [44]. Importantly, the Commission recommends direct body fat measurement tools such as DXA as core diagnostic components. Our use of DXA-derived VAT and A/G ratio thus offers practical validation of this model and supports its application in real-world settings, particularly in resource-equipped clinical environments [44].

In line with this emerging perspective, we propose that clinicians incorporate regional adiposity markers into routine evaluation protocols, particularly for peri- and postmenopausal women. Targeted DXA screening may be especially warranted for women over 50 years of age, even when BMI is within the normal range. In low-resource settings where DXA is unavailable, bioelectrical impedance analysis (BIA)—as a low-cost and widely accessible method—may offer a viable alternative for estimating visceral fat and body composition with greater precision than traditional anthropometric indices [45,46]. Waist circumference also remains a useful surrogate marker for central fat accumulation [47,48,49].

Furthermore, these findings support the need to develop population-specific guidelines that consider cultural and ethnic variations in body composition. For example, the Korean Society for the Study of Obesity (KSSO) defines overweight as BMI ≥ 23 kg/m^2^ and obesity as BMI ≥ 25 kg/m^2^, thresholds that better reflect the elevated cardiometabolic risk in Asian populations compared to Western criteria [32]. Additionally, these findings highlight the need for further epidemiological research to define population-specific thresholds for regional adiposity indices—such as VAT area and A/G ratio—and to clarify their associations with cardiovascular and metabolic outcomes across different ethnic and geographic populations.

Together, our findings advocate for a more nuanced, function-oriented framework for obesity diagnosis and risk stratification in women across midlife and aging, consistent with both international and national recommendations.

### 4.2. Study Limitations: Methodological, Demographic, and Measurement Considerations

This study has several limitations that must be acknowledged. First, the retrospective cross-sectional design precludes causal inference regarding the relationship between age and adiposity changes. Second, the sample size was unevenly distributed across age groups, with a markedly smaller number of participants aged ≥ 70 years. This imbalance may have limited statistical power and increased variability in estimates for the oldest group. Although our primary aim was to assess overall trends across midlife and aging, the interpretation of findings in the ≥70 group should be made with caution. Third, menopausal status was not directly measured and was instead inferred based on age, which may have led to misclassification. Fourth, while participants were recruited from both a university hospital and a regional medical center, the cohort may not be fully representative of the general Korean female population. Cultural, behavioral, and environmental factors unique to this population—such as dietary patterns, physical activity, and healthcare access—may limit generalizability to other populations. Moreover, as the entire cohort consisted exclusively of Korean women, our findings may not be generalizable to non-Asian populations. Prior studies have demonstrated significant ethnic differences in fat distribution patterns and in the relationship between BMI and body fatness, particularly between Asian and Western populations. Therefore, caution is warranted when extrapolating these results to ethnically diverse or global populations. Fifth, although DXA is a validated method for body composition analysis, its estimation of visceral fat remains indirect compared to gold-standard imaging modalities such as CT or MRI. Lastly, several potential confounders—including socioeconomic status, dietary intake, physical activity, and hormone therapy use—were not assessed and could have influenced the observed adiposity patterns. Future prospective studies with larger and more balanced sample sizes across age groups, as well as the direct assessment of menopausal status and inclusion of diverse populations, are warranted to validate and extend these findings.

### 4.3. Study Strengths: Comprehensive DXA-Based Assessment and Clinical Relevance

This study has several notable strengths. It included a relatively large and age-diverse sample of 917 Korean women aged 40 years and older, allowing for robust comparisons across age groups. Second, we utilized standardized whole-body DXA measurements to assess not only general adiposity indices such as BMI and total body fat percentage (TB%F), but also regional fat distribution markers including fat mass index (FMI), visceral adipose tissue (VAT), and the android-to-gynoid fat ratio (A/G ratio). This comprehensive approach enhances the precision of body composition assessment and highlights the limitations of BMI-based obesity classification. This study provides valuable population-specific data on Asian women, particularly on Korean women, a group underrepresented in existing DXA research. Lastly, our findings emphasize the presence of metabolically unfavorable fat distribution—such as excess VAT and elevated A/G ratio—even in women with normal BMI, thereby underscoring the need for improved risk stratification methods beyond BMI.

Importantly, to the best of our knowledge, this is the first study to focus specifically on adiposity changes from the perimenopausal to postmenopausal period in Korean women using DXA-derived indices. By identifying progressive central fat accumulation during this transitional stage, our findings offer clinically relevant insights for the early detection of cardiometabolic risk in aging women.

## 5. Conclusions

This study highlights distinct age-related trends in body fat distribution among Korean women aged 40 years and older. While general adiposity measures plateau in later life, central adiposity—as reflected by increasing VAT and A/G ratio—continues to rise with age. A significant proportion of women with normal BMI exhibited excess visceral fat and unfavorable fat distribution, indicating the limitations of BMI in detecting metabolically relevant adiposity. These results underscore the clinical value of incorporating DXA-derived indices into obesity assessment to more accurately identify women at elevated cardiometabolic risk. Comprehensive body composition evaluation may contribute to earlier identification and intervention in aging women, ultimately improving long-term health outcomes. Our findings support the integration of regional fat distribution indices into clinical screening protocols, particularly for midlife and older women who may be misclassified by BMI alone.

## Figures and Tables

**Table 1:** General Characteristics of Study Participants by Age Group.

Age Group (Years)	*n*	Mean Age (Years)	Mean Weight (kg)	Mean Height (cm)	Mean BMI (kg/m^2^)
40–49	128	46.9	55.5	159.8	21.7
50–59	545	54.7	56.5	158.4	22.8
60–69	209	63.1	56.2	156.3	23.6
≥70	35	72.7	54.2	154.2	22.8
Total	917	56.2	56.2	157.7	22.8

BMI, body mass index.

**Table 2:** DXA-Derived Adiposity Indices by Age Group.

Age Group (Years)	BMI (kg/m^2^)	TB%F (%)	FMI (kg/m^2^)	A/G Ratio	VAT (cm^2^)
40–49	21.7	38.2	8.34	0.93	87.7
50–59	22.8	39.0	8.85	1.09	99.6
60–69	23.6	38.6	8.72	1.01	102.2
≥70	22.8	38.4	8.64	1.06	107.6
Total (Mean ± SD)	22.8 ± 7.2	38.8 ± 4.8	8.74 ± 2.38	1.05 ± 1.82	98.8 ± 40.4

BMI, body mass index; TB%F, total body fat percentage; FMI, fat mass index; A/G ratio, android-to-gynoid fat ratio; VAT, visceral adipose tissue area.

**Table 3:** Prevalence of Excess Adiposity According to DXA-Based Indices by Age Group.

Age Group (Years)	BMI ≥ 23 (%)	TB%F ≥ 40% (%)	FMI ≥ 9 kg/m^2^ (%)	A/G Ratio > 1.0 (%)
40–49	28.9	33.6	28.9	31.3
50–59	38.7	44.6	37.4	50.1
60–69	44.5	39.7	43.5	55.9
≥70	42.8	42.9	37.4	60.0
Total	38.8	41.9	37.6	49.2

BMI represents the combined proportion of individuals classified as overweight (BMI 23.0–24.9 kg/m^2^) and obese (BMI ≥ 25.0 kg/m^2^), based on WHO Asia-Pacific and KSSO criteria. BMI, body mass index; TB%F, total body fat percentage; FMI, fat mass index; A/G ratio, android-to-gynoid fat ratio; VAT, visceral adipose tissue area.

**Table 4:** Adiposity Distribution by BMI Category.

BMI Category	TB%F (Mean ± SD)	FMI (Mean ± SD)	VAT			Apple Shape (%)	Pear Shape (%)
			Normal >100 (%)	Borderline 100–160 (%)	High Risk ≤160 (%)		
Underweight	32.2 ± 3.7	5.6 ± 0.8	100	0	0	8.3	91.7
Normal	37.3 ± 3.9	7.9 ± 2.2	78.0	21.6	0.4	35.7	64.3
Overweight	40.5 ± 3.7	9.5 ± 1.0	31.2	62.8	6.0	67.3	32.7
Obese ≥ 25	43.0 ± 3.5	11.3 ± 1.3	7.6	55.4	36.9	82.8	17.2
Severe Obese ≥ 30	47.9 ± 10.1	13.6 ± 2.8					

BMI, body mass index; TB%F, total body fat percentage; FMI, fat mass index; VAT, visceral adipose tissue area.

## Data Availability

The datasets generated during and/or analyzed during the current study are available from the corresponding author on reasonable request.

## Supplementary Materials

The following supporting information can be downloaded at: https://www.mdpi.com/article/10.3390/medicina61071301/s1, Table S1: The STROBE checklist
