# Supplementary material for: Age-Related Trends in Dual-Energy X-Ray Absorptiometry–Measured Adiposity and Their Clinical Relevance: A Multicenter Cross-Sectional Study of Korean Peri- and Postmenopausal Women
Source: Medicina (Kaunas). 2025 Jul 19;61(7):1301. doi: 10.3390/medicina61071301 (PMC12298740; doi:10.3390/medicina61071301)
Supplement: Supplementary file 1 [file medicina-61-01301-s001.zip › medicina-3723702-supplementary.pdf]

STROBE Checklist for Cross-Sectional Studies

Title of Study: Age-Related Trends in DXA-Measured Adiposity and Their Clinical Relevance in Korean Peri- and Postmenopausal Women

Study Design: Multicenter retrospective cross-sectional study

Corresponding Author: Dr. Jae-yen Song

Item No	Recommendation	Addressed in Manuscript
1a	Indicate the study's design in the title or abstract	Abstract and title include "cross-sectional" and "DXA-based study"
1b	Provide an informative and balanced summary in the abstract	Revised abstract includes background, methods, results, and clinical relevance
2	Explain background/rationale	Introduction paragraph 1–3
3	State specific objectives and hypotheses	Final paragraph of Introduction clearly states primary and secondary objectives and the research question
4	Present key elements of study design	First paragraph of Methods
5	Describe setting, locations, and relevant dates	Methods: two Korean hospitals, 2018–2021
6a	Eligibility criteria and participant selection	Methods: inclusion/exclusion criteria provided
7	Define all outcomes, exposures, confounders	Methods: adiposity indices, BMI, and thresholds clearly defined
8	Data sources and measurement methods	Methods: DXA protocol, calibration, and device info specified
9	Address bias	Mentioned standardization and calibration across centers to minimize measurement bias
10	Study size	Number of participants specified; retrospective analysis of available records for 914 patients e
11	Handling of quantitative variables	ANOVA, threshold cutoffs described
12a–e	Statistical methods, subgroup analysis, missing data	Methods: ANOVA with Bonferroni, significance level, no missing data imputation noted
13a–c	Participant flow	Numbers by age group mentioned in Results; no flow diagram used
14a–b	Descriptive data	Tables 1–2 present detailed data; missing values not significant
15	Outcome data	Table 3–4 and narrative results
16a–c	Main results, confounder-adjusted estimates	Raw percentages presented; BMI stratified analysis; clinical implications discussed

17	Other analyses (e.g., subgroups)	Results include BMI subgroup misclassification rates
18	Key results aligned with objectives	Discussion first paragraph
19	Limitations discussed	Separate Limitations section addresses methodology, generalizability, and confounding
20	Interpretation considering evidence	Discussion last 2 paragraphs
21	Generalizability discussed	In Limitations and Conclusion
22	Source of funding and role of funders	Added in Acknowledgments section of revised manuscript
